# Paeoniflorin reduce *luxS*/AI-2 system-controlled biofilm formation and virulence in *Streptococcus suis*

**DOI:** 10.1080/21505594.2021.2010398

**Published:** 2021-12-18

**Authors:** Jinpeng Li, Qingying Fan, Manyu Jin, Chenlong Mao, Hui Zhang, Xiaoling Zhang, Liyun Sun, Daniel Grenier, Li Yi, Xiaogai Hou, Yang Wang

**Affiliations:** a College of Animal Science and Technology, Henan University of Science and Technology, Luoyang, China; b Key Laboratory of Molecular Pathogen and Immunology of Animal of Luoyang, Luoyang, China; c China Animal Health and Epidemiology Center, Qingdao, China; d Groupe de Recherche En Écologie Buccale (Greb), Faculté de Médecine Dentaire, Université Laval, Quebec City, Canada; e College of Life Science, Luoyang Normal University, Luoyang, China; f College of Agriculture/College of Tree Peony, Henan University of Science and Technology Luoyang China

**Keywords:** Paeoniflorin, *Streptococcus suis*, LuxsS/Ai-2 system, biofilm, virulence factor

## Abstract

*Streptococcus suis* (*S. suis*), more specifically serotype 2, is a bacterial pathogen that threatens the lives of pigs and humans. Like many other pathogens, *S. suis* exhibits quorum sensing (QS) system-controlled virulence factors, such as biofilm formation that complicates treatment. Therefore, impairing the QS involving LuxS/AI-2 cycle in *S. suis*, may be a promising alternative strategy for overcoming *S. suis* infections. In this study, we investigated paeoniflorin (PF), a monoterpenoid glycoside compound extracted from peony, as an inhibitor of *S. suis* LuxS/AI-2 system. At a sub-minimal inhibitory concentration (MIC) (1/16 MIC; 25 μg/ml), PF significantly reduced biofilm formation by *S. suis* through inhibition of extracellular polysaccharide (EPS) production, without affecting bacterial growth. Moreover, evidence was brought that PF reduces AI-2 activity in *S. suis* biofilm. Molecular docking indicated that LuxS may be the target of PF. Monitoring LuxS enzymatic activity confirmed that PF had a partial inhibitory effect. Finally, we showed that the use of PF in a mouse model can relieve *S. suis* infections. This study highlighted the anti-biofilm potential of PF against *S. suis*, and brought evidence that it may as an inhibitor of the LuxS/AI-2 system to prevent *S. suis* biofilm-related infections. PF can thus be used as a new type of natural biofilm inhibitor for clinical application.

## Introduction

*Streptococcus suis* (*S. suis*) is one of the most important zoonotic pathogens that mainly colonizes the upper respiratory of pigs, leading to sepsis, meningitis, pneumonia, and toxic shock-like syndrome [1,2]. *S. suis* not only causes huge economic losses to the pig industry, but as a zoonotic pathogen also poses a threat to human public health [3]. In China, Vietnam, and Thailand, *S. suis* has caused thousands of human diseases and has been identified as one of the culprits of human bacterial meningitis [4]. Studies have found that *S. suis* small RNA rss04 can help induce meningitis by regulating the synthesis of capsular and inducing biofilm formation in mouse infection model [5]. The route of intracranial subarachnoid infection in mouse infection model also further confirms the important role of *S. suis* biofilm in meningitis [6]. The above-mentioned research clearly shows that the biofilm of plays a key role in *S. suis* meningitis. In addition, the ability of *S. suis* to form biofilms in the host causes persistent infections difficult to eradicate with antibiotics [7], and inhibit the formation of extracellular traps of neutrophils [8]. In fact, it is estimated that approximately 65% of hospital infections and up to 75% of bacterial infections that occur in the human body are related to biofilms.

Biofilm formation in bacteria relies on the QS system, a “language” for bacterial communication, which can regulate the activities of bacteria to adapt to the harsh external environment [9]. LuxS/AI-2 is a QS system discovered in *S. suis* serotype 2 by our group [10]. The core of LuxS/AI-2 system is the protease LuxS involved in the formation of the signal molecule AI-2, which is a by-product of methyl metabolism. Research in *Vibrio harveyi* (*V. harveyi*) indicated that AI-2 can affect the ability of bacterial biofilm formation by inhibiting the expression of type III secretion system genes [11]. Previous work by our research group showed that the adhesion, gene expression, and virulence of *S. suis* in a biofilm state differ from the planktonic state, and it is speculated that the virulent strains of *S. suis* may rely on the formation of biofilm to achieve their infectivity and their ability to exhibit drug resistance [12–15].

Given the ability of bacteria to develop resistance to traditional antibiotics, it is particularly important to identify/develop antibacterial agents exhibiting novel antibacterial modes of action. A promising strategy is based on the inhibition of biofilm formation by pathogens. Recent studies have shown that natural products have advantages over traditional antibiotics because of their ability to regulate the formation of biofilms by bacterial pathogens [16,17]. The benefits of natural products in inhibiting biofilms are related to their high specificity and low toxicity [18]. Plants are the most extensive source of natural products, some of which have been found to exhibit anti-biofilm properties. For instance, cranberry proanthocyanidins have been reported to interfere with the quorum sensing of bacteria by competing with the binding of spontaneous inducers and receptors [19]. Similarly, extracts from green tea and onion can suppress the biofilm by interfering with the bacterial quorum sensing system [20,21]. In addition, the sub-minimum inhibitory concentration (sub-MIC) of rhubarb water extract can inhibit the biofilm of *S. suis* by inhibiting the histidine kinase and the two-component signal transduction systems (TCSs) constituent proteins of histidine kinase and response regulator. However, plant extracts as new biofilm inhibitors also have some drawbacks, such as toxicity, reactivity and instability, and the effective ingredients of the extract are often not clear [22]. Given that numerous plants are edible and considered safe, it is necessary to continue to search for potential anti-biofilm drugs.

Paeoniflorin (PF) is a monoterpene glycoside compound found in many *Paeoniaceae* plants such as peony, for which various pharmacological effects including antibacterial, antioxidant, anti-inflammatory, and anti-tumor have been identified [23]. Studies have found that PF can inhibit the formation of carbapenem-resistant *Klebsiella pneumoniae* (CRKP) biofilm and have a significant inhibitory effect on CRKP [24].

In this work, we provide evidence that PF inhibits the formation of *S. suis* biofilm and its virulence in a mouse model by affecting the synthesis of AI-2 signaling molecule of the LuxS/AI-2 system. An in-depth analysis of the biofilm inhibitory mechanism of PF at the molecular level was also performed in view to develop a new anti-*S. suis* biofilm inhibitor.

## Materials and methods

### Bacterial strains, growth conditions, and reagents

*S. suis* HA9801, *Vibrio harveyi* BB120, and *V. harveyi* BB170 were used to investigate the anti-biofilm activities of PF. *S. suis* HA9801 is a virulent serotype 2 strain isolated from a diseased swine in the HaiAn City in 1998. A *luxS* mutant (Δ*luxS*) of *S. suis* and a complemented mutant strain (CΔ*luxS*) were constructed in our previous study [10]. *S. suis* was grown in Todd Soy broth (TSB) at 37°C or plated on TSB agar. *Escherichia coli* (*E. coli*) BL21 (DE3) was transformed with pET28-luxS in our previous study [25]. The pET28-luxS (DE3) was grown at 37°C in LB medium supplemented with 50 μg/mL of kanamycin. *V. harveyi* BB120 and *V. harveyi* BB170 were kindly provided by Professor XianGan Han from Shanghai Veterinary Research Institute Chinese Academy of Agricultural Sciences (Shanghai, China). *V. harveyi* was grown in autoinducer bioassay (AB) medium at 28°C. PF (CAS: 23,180–57-6, HPLC purity ≥99.5%) was obtained from the 3B Scientific Corporation Limited (Wuhan, Hubei, China). PF was transferred to pre-weighed vials and stored at −20°C. Prior to be used, PF was dissolved in distilled water and filter-sterilized. The AI-2 precursor molecule, (S)-4,5-Dihydroxy-2,3-pentandione (DPD), was purchased from Omm Scientific Inc. (Dallas, TX) and used at a concentration of 3.9 μM.

### Growth kinetics of S. suis

Growth kinetics was operated using the previously procedure with slight improvement [12]. Briefly, after determining the minimum inhibitory concentration (MIC) and minimum bactericidal concentration (MBC) (Supplementary materials 1), *S. suis* HA9801 was grown at sub-MIC (1/2 MIC, 200 μg/ml; 1/4 MIC, 100 μg/ml; 1/8 MIC, 50 μg/ml; 1/16 MIC, 25 μg/ml; 1/32 MIC, 12.5 μg/ml) in a 96-well microtiter plate at 37°C. Samples were collected every hour, and colony-forming units (CFU) were determined by plating on TSA medium.

### Biofilm inhibition assay

Biofilm formation ability of *S. suis* was monitored as described previously [26]. *S. suis* HA9801 was grown in TSB medium 12 h at 37°C, and then the bacterial culture was diluted with fresh TSB medium to a concentration of 10^6^ CFU/ml for the anti-biofilm assay. A PF stock solution was freshly prepared in distilled water at a concentration of 1.6 mg/ml. After filtering with a 0.22 water-based filter, the stock solution was diluted at different concentrations ranging from 6.25 to 25 μg/ml in sterile culture medium, and an equal volume was added to the above bacterial suspension and incubated at 37°C for 24 h without shaking. A control culture with *S. suis* and no PF was also performed. Following growth, planktonic bacteria were removed and the biofilm was stained with 1% crystal violet for 10 min and then washed with phosphate-buffered saline (PBS). After adding 95% ethanol to release the dye, the absorbance at OD595 nm was recorded with a Tecan GENios Plus microplate reader (Tecan, Austria). Biofilm formation by the Δ*luxS* mutant of *S. suis* HA9801, previously constructed by our research group [10], was assessed as described above. The assay was performed in the presence of DPD (final concentration of 3.9 μM) and PF at 25 μg/ml. A control culture with Δ*luxS* mutant and no PF was also performed. All assays were performed in triplicate and repeated three times.

### Scanning electron microscopy of biofilms

Biofilms were observed for the following cultures: *0suis* HA9801; *S. suis* HA9801 + 25 μl/ml PF; Δ*luxS* strain; Δ*luxS* + 25 μl/ml PF + AI-2 (3.9 μM DPD). An overnight growth *S. suis* was diluted to reach a concentration of 10^5^–10^6^ CFU/ml. Then, the culture of 1 mL was added to a 24-well microplate (In vitro scientific, Hang Zhou, China) containing a sterile cell slide (0.5 cm^2^). After culturing for 24 h at 37°C, the cell slide were rinsed with sterile PBS (0.2 M, PH = 7.2) so as to remove planktonic and loosely-bound bacteria.

The biofilms were treated with 2.5% (w/v) glutaraldehyde for 6 h, washed with PBS (0.2 M, PH = 7.2), and fixed in 1% osmium tetroxide. And subjected to dehydration in a gradient alcohol system (25, 40, 55, 75, 90, and 100% ethanol). The samples are handled carefully throughout the drying process to prevent damage to the biofilm. And gold sputtered with a sputter coater (10 mA, 3 min) and observed by SEM (JSM-5610LV, Japan).

### Confocal laser scanning microscopy of biofilms

Biofilm fromed by *S. suis* HA9801, *S. suis* HA9801 + 25 μl/ml PF, Δ*luxS* strain, and Δ*luxS* + 25 μl/ml PF + AI-2 (3.9 μM DPD) were also examined by Confocal Laser Scanning Microscopy (CLSM) (Carl Zeiss LSM800, Germany). Biofilms were formed in a 24-well microplate containing a round coverslip according to the above method. Following growth, the round coverslips were gently washed three times in PBS to remove planktonic and loosely-attached bacteria. After drying at normothermic, the biofilms were labeled with SYTO 9 according to the manufacturer of the LIVE/DEAD BIOFILM kit (ABI L10316, Invitrogen, USA). The stained biofilms were observed by CLSM equipped and magnification at 630 × .

### Capsular polysaccharide formation assay

A 10-mL overnight culture of *S. suis* (HA9801, Δ*luxS*) was used to inoculate 990 mL of TSB medium supplemented with PF at a final concentration of 25 μg/ml, and the culture was incubated at 37°C for 24 h. Control cultures with no PF were also prepared. After centrifugation at 10,000 g, the bacterial pellets were suspended in 10 mL of glycine buffer (0.1 M, pH 9.2), and then 100 mg of crystalline salt-free egg white lysozyme was added. The bacterial suspensions were incubated at 37°C for 6 h with shaking (100 rpm/min). After centrifugation at 10,000 g, proteinase K at a final concentration of 100 μg/ml was added to the supernatant, and incubation was carried out at 55°C for 2 h. CaCl_2_ at a final concentration of 0.1 M was added, and the solution was stirred for 1 h prior to add 25% (v/v) absolute ethanol. After 2 h at 4°C, the solution was centrifuged at 8000 g. To the supernatant, 80% (v/v) absolute ethanol was added and the solution was kept overnight at 4°C, prior to centrifuge (8000 g, 4°C) to harvest capsular polysaccharides (CPS). The CPS was quantified by the previously described method using phenol-sulfuric acid [27]. The change rate of CPS was calculated according to the following equation: Change rate (%) = 100% × (carbohydrate content of sample group – carbohydrate content of *S. suis* group)/carbohydrate content of *S. suis* group.

### Extracellular polysaccharide (EPS) formation assay

The effect of PF on EPS formation by *S. suis* and Δ*luxS* mutant strain was determined by a previously described method [28]. TSB medium supplemented with PF at a final concentration of 25 μg/mL was inoculated with an overnight inoculum (1%) of of *S. suis* (HA9801, Δ*luxS*). After incubation at 37°C for 24 h, 1 ml of the culture centrifuged for 10 min (12,000 g, 4°C), and the supernatant was filtered (0.22 μm aqueous filter) prior to add 3 mL of pre-cooled ethanol, and let stand at 4°C for 24 h. The solution was then centrifuged (10 min, 12,000 g, 4°C) and the pellet harvested, which contains EPS was suspended in 1 mL of deionized distilled (dd) water. Using glucose as the standard, the EPS content was determined by the phenol-sulfuric acid method [27]. The equation for measuring the standard curve is y = 0.0581x + 0.0913 (R^2^ = 0.9969). The change rate of CPS was calculated according to the following equation: Change rate (%) = 100% × (carbohydrate content of sample group – carbohydrate content of *S. suis* group)/carbohydrate content of *S. suis* group.

### Real-time RT-PCR

RNA was obtained from cultures of WT, WT+PF and Δ*luxS* groups using the Bacterial Total RNA Isolation Kit (G710KA6220, Sangon Biotech, China) following the Kit instructions. Samples was assessed. Subsequently, the RNA samples of WT, WT+PF and Δ*luxS* were converted into cDNA using Reverse Transcription Kit with dsDNase (70,060,200, Biosharp, China). The cDNA samples of WT, WT+PF and Δ*luxS* was amplified by the Unlversal SYBR qPCR Master Mix (100,010,629,049, Biosharp, China). The amplification system and PCR program according to the instruction manual of the kit. The reference gene is 16S rRNA (Supplementary materials 2).

### AI-2 activity assay

To determine the effect of PF on the activity of AI-2 [29], *S. suis* HA9801 and Δ*luxS* were grown overnight at 37°C, the bacterial cultures were diluted to 10^5^ CFU/ml, and divided into three test groups: *S. suis; S. suis* with 25 μg/ml PF; Δ*luxS*. The above-mentioned diluted bacterial cultures were incubated at 37°C for 12 h. During this period, 1-ml aliquots of the cultures were harvested at 4, 6, 8, 10, and 12 h, and centrifuge at 10,000 g for 10 min at 4°C. The negative control was the supernatant obtained by centrifugation of *E. coli* DH5α under the above culture conditions. The supernatants were filtered through a 0.22 μm filter and stored at −80°C.

In order to detect AI-2 activity in each test group, *V. harveyi* BB170 cultured overnight at 28°C was diluted 5000 times with AB medium. Ninety μL of the BB170 diluted culture along with 10 μL of AI-2 supernatants (prepared from the above test group) were incubated at 28°C in the dark for 6 h, and the bioluminescence value was measured using a Promega Luminometer at a wavelength of 490 nm. The test was repeated 3 times independently. The test results are displayed in the form of ratio: luminescence value of each test group/luminescence value of *E. coli* DH5α.

### Molecular docking assay

As previously described [30], a virtual molecular docking analysis was conducted to determine how PF interacts with the LuxS protein. The chemical structure of PF was downloaded from PubChem, while the three-dimensional structure of LuxS protein of *S. suis* HA9801 was previously reported by our group. The LuxS protein model was pre-processed with the SYBYL-X 2.1 software (Triops, USA), including hydrogenation, side-chain repair, and deletion of water molecules. Finally, the AMBER force field was used to minimize the energy of the protein. PF was operated by adding hydrogen atoms and Gasteiger-Hückel charge, and optimized with the Tripos force field of SYBYL-X 2.1 software (convergence criterion: 0.005 kcal/(Å mol)), and saved in MOL2 format.

### Inhibition assay of PF on LuxS enzyme activity

The LuxS protein was purified from BL21 competent cells transformed with the plasmid pET28-*luxS* as previously reported by our group [25]. The LuxS protein expression vector was grown in LB medium containing 50 μg/ml kanamycin at 37°C to a 0.6 at OD_600 nm_. Then, 0.1 mM isopropyl-β-D-thiogalactopyranoside (IPTG) was added to induce LuxS expression and the bacterial culture was further incubated at 37°C for 5 h. The cells were collected by centrifugation (12,000 g, 4°C), suspended in lysis buffer, incubated at 25°C for 10 min, and lysed by an ultrasonic treatment (working power: 400 W; 80 cycle of 5 s with rest of 10 s between each cycle). The lysate was centrifuged (40,000 g, 4°C) for 30 min, and the supernatant was retained after filtration (0.45 µm pore size). The clarified lysate was loaded onto a 1 ml HisTrap HP column (GE Healthcare Life Sciences) using an GE Akta Pure (GE Healthcare Life Sciences). Proteins were eluted from the column using a linear gradient of elution buffer (20 mM Tris–HCl pH 8.0, 300 mM NaCl, 1 M imidazole). Protein concentration was determined using a bicinchoninic (BAC) protein assay (Supplementary materials 3) [25].

The preparation method of LuxS substrate (SRH) was based on a previously published report [31]. Briefly, SAH (Sigma) was dissolved in 1 M HCl at a concentration of 1 mg/ml in a boiling water bath for 20 min. Then, the pH was adjusted to 7.2 with 1 M NaOH, and the SRH solution was diluted in a 200 mM sodium phosphate buffer (pH 7.2) at a concentration of 4 mM. LuxS activity was determined by quantification of homocysteine by the Ellman method [32]. LuxS reaction (total volume = 100 μl) contained 0.5 mM EDTA, 200 mM (pH 7.2) sodium phosphate buffer and 20 μg/ml LuxS. The reaction was initiated by adding different concentrations of SRH (1–1000 μM) and performed at 37°C for 5 min. Finally, 100 μl of 2 mM 5,50-dithiobiguanide (2-nitrobenzoic acid) was added, and the mixtures were further incubated at 37°C for 10 min. The absorbance at 412 nm was monitored with a Synergy HT Multi-Detection Reader (BioTek Instruments, USA). Then, the standard curve (Y = 0.01300*X + 0.006456 R^2^ = 0.9997) was used to calculate the homocysteine concentration (Figure S1). Km (LuxS) value was determined by Michaelis–Menten equation nonlinear equation using graphPad 9.0. According to the above experimental method, different concentrations of PF (6.25, 12.5, 25 μg/ml) were added to the reaction mixtures, and the Km values were determined.

### Cell viability assay

Cell viability was detected using a previously described protocol [33]. Briefly, human laryngeal epidermoid carcinoma (HEp-2) cells were cultured in RPMI 1640(PM150110, Procell, China) containing 10% bovine serum (16,170,060, Thermo Fisher, USA) and were seeded (1 × 10^4^ cells) into the wells of a 96-well microplate and allowed to adhere for 24 h at 37°C under 5% CO_2_. The cells were treated with two-fold serial dilutions of PF (25, 50, 100, 200, and 400 μg/ml) for 10 min. Then, PF was washed away and fresh culture medium was added prior to further incubate for 24 and 48 h. Cell viability was determined using an MTT (3-[4,5-diethylthiazol-2-yl]-2,5diphenyltetrazolium bromide) colorimetric assay according to the manufacturer’s protocol. The cell survival rate is expressed as a percentage of the control value.

### Mouse protection assay

A mouse protection assay was performed according to a protocol previously published by our group [15]. Mice were intraperitoneally injected with 1 × 10^6^ CFU/ml of *S. suis* to induce infection. The mice infected with *S. suis* were randomly divided into five groups, each with 10 mice. The protective treatment administrated by injection of 100 µl through the tail vein consisted in: Group 1: WT + solvent (distilled water) group; Group 2: WT + 25 μg/g PF; Group 3: WT + 50 μg/g PF; Group 4: WT + 100 μg/g PF; Group 5: Δ*luxS* + solvent. The first administration was 2 h after the establishment of the *S. suis* infection, and thereafter twice a day. The mortality of the mice was recorded after 7 days.

### Mouse anti-infection assay

*S. suis* was cultured overnight at 37°C, and then diluted with sterile PBS to a concentration of 5 × 10^6^ CFU/mL. Fifteen female Balb/c mice (4–6 weeks) were equally divided into four groups, one of which was a blank control group without any treatment, and a group was inoculated with 200 μl of Δ*luxS* by intraperitoneal injection. Each mouse in the other two groups was inoculated with 200 μl of *S. suis* HA9801 by intraperitoneal injection. Then, different concentrations of PF (0, 100 μg/g) were used for treatment via tail vein injection. The first treatment was two hours after the infection of *S. suis*, and then two treatments a day thereafter. All mice were sacrificed two days later, and the brain, lung, liver, and spleen were dissected.

Part of the brain, lung, liver, and spleen were aseptically taken and fixed in 4% paraformaldehyde (pH = 7) for 24 h. The tissues were embedded in paraffin, cutted into 4 μm-thick sections, stained with hematoxylin and eosin, and observed with an optical microscope. The remaining brain, lung, liver, and spleen tissues were added into 1 ml of PBS, homogenized, and serially diluted. The bacterial CFU counts were then determined.

### Statistical methods

**Figure 1.**
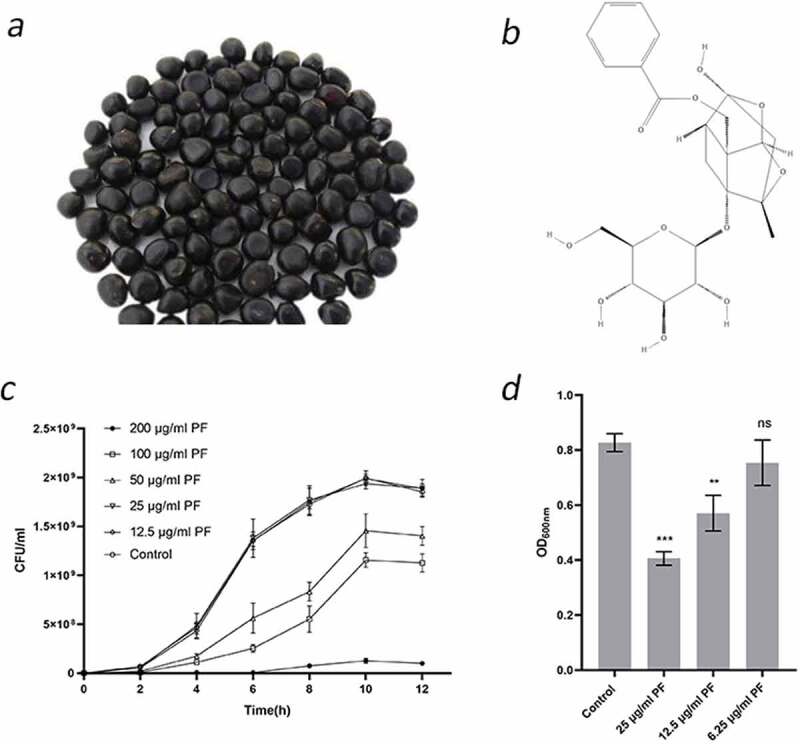
Effects of sub-inhibitory concentrations of PF on *S. suis* growth and biofilm formation. (a) Peony seeds. PF used in this study was extracted from peony seed cake. (b) Two-dimensional structure of PF. (c) Growth curve of *S. suis* HA9801 in the presence of PF. Growth was monitored by determination of CFU at the time points indicated. (d) Biofilm formation by *S. suis* HA9801 in the presence of sub-inhibitory concentrations of PF. Biofilm was quantified by crystal violet staining following bacterial growth. Data are shown as the mean ± SD of triplicate experiments. Statistical significance was assessed by unpaired Student’s two-sided t-test compared to the control group. ** p < 0.01, *** p < 0.001

**Figure 2.**
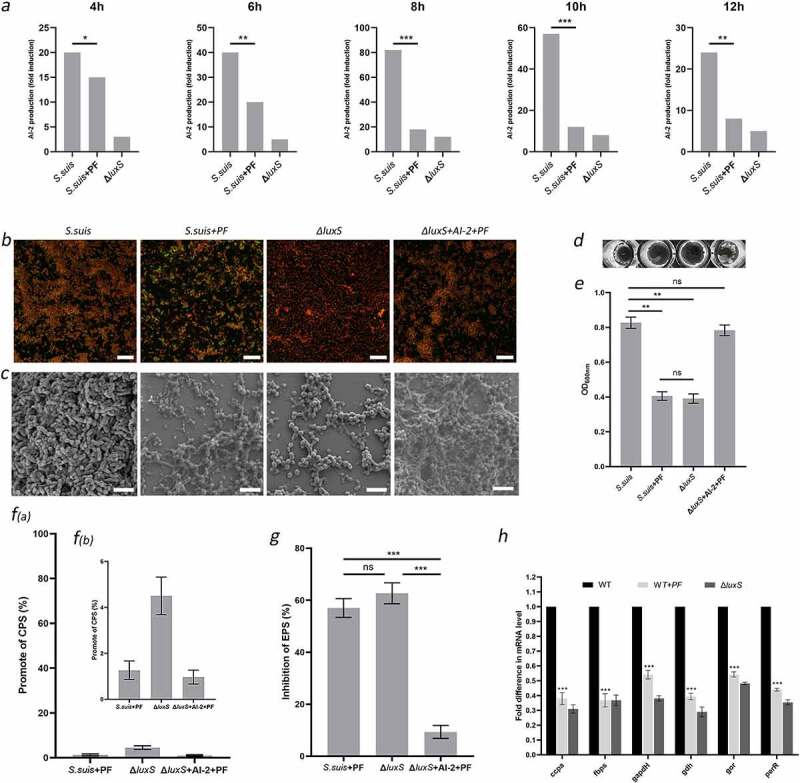
PF reduces biofilm formation in *S. suis* by weakening the extracellular polysaccharide matrix through the LuxS/AI-2 system. (a) AI-2 sproduction by *S. suis* HA9801, *S. suis* + PF (25 μg/ml), and Δ*luxS*. (b) Confocal laser scanning microscopy of *S. suis* biofilms; scale bars: 20 µm. (c) Scanning electron microscopy of *S. suis* biofilms; scale bars: 5 µm. (d) and (e) Crystal violet-stained *S. suis* biofilms. (f) Effect of PF on *S. suis* capsular polysaccharide production. (g) Effect of PF on *S. suis* extracellular polysaccharide production. (h) Relative expression of virulence genes by the *S. suis*. The gene expression level for the wild type strain (HA9801) in the absence of PF was set at 100%, and the gene expression level for the wild type strain + PF (25 μg/ml) and Δ*luxS* were relative to that of the wild type strain. In figures (a), (e), (f), (g), and (h), data are shown as the mean ± SD. Statistical significance was assessed by unpaired Student’s two-sided t-test compared to the control group. ** p < 0.01, *** p < 0.001. All experiments were performed in triplicate

The significance of the data in Figure 1d, 2a, 2e and 2g was analyzed according to unpaired Student’s two-sided t-test. *p < 0.05, **p < 0.01, and ***p < 0.001. The samples/animals were randomly allocated to experimental groups and processed for blind evaluation.

## Results

### Anti-biofilm properties of PF

MIC and MBC values of PF against *S. suis* HA9801 were 400 and >1600 μg/ml, respectively. Then, we used the CFU method to analyze the effect of sub-inhibitory concentrations of PF on the growth kinetics of *S. suis*. As shown in Figure 1c, when the concentration of PF ≤ 25 μg/ml (1/16 MIC), the growth of *S. suis* was not significantly different from that of the control (no PF). Furthermore, the semi-quantitative determination of biofilm by crystal violet staining showed that PF at 12.5 and 25 μg/ml significantly inhibits biofilm formation by *S. suis*, while a concentration of 6.25 μg/ml had no biofilm inhibitory effect.

### PF affects EPS production through LuxS/AI-2 system for anti-biofilm activity

In order to identify the mechanism by which PF affects the formation of biofilm in *S. suis*, we monitored changes in the amounts of AI-2 secreted when bacterial growth was achieved in the presence of PF. As shown in Figure 2a, compared with control growth (no PF), the amount of AI-2 signal molecule secreted by *S. suis* HA9801 was significantly reduced for growth in the presence of PF, as also observed for the growth of Δ*luxS* (in the absence of PF). The above suggests that PF modulates the production of the signal molecule AI-2 by the LuxS/AI-2 system or/and directly acts on the AI-2 molecule during the growth process. To verify this hypothesis, we monitored biofilm formation by Δ*luxS* in a culture medium containing PF and supplemented with AI-2. As shown in Figure 2e, there was no significant difference between the biofilm formation ability of the wild-type strain (HA9801) grown in the presence of PF (25 μg/ml) and the Δ*luxS* mutant (no PF). Moreover, adding the AI-2 signal molecule to Δ*luxS* grown in the presence of PF (25 μg/ml), restored its biofilm formation ability, which was not significantly different from that of the *S. suis* wild strain. Biofilms formed under the above conditions were observed by laser confocal microscopy (Figure 2b) and scanning electron microscopy (Figure 2c). The *ΔluxS* strain grown in the presence of PF and AI-2 formed a dense biofilm (Figure 2b) with a three-dimensional structure composed of bacteria and biofilm matrix, with channels for allowing nutrient exchange with the external environment, similar to that formed by the wild strain (Figure 2c). On the contrary, the biofilms formed by the wild strain in the presence of PF and the Δ*luxS* mutant were similar; the bacterial cells were more dispersed and aggregated less, and the biofilm matrix was greatly weakened. These results indicate that PF can weaken the biofilm matrix through LuxS/AI-2 system, but will not inactivate AI-2. Since the biofilm matrix is mainly composed of polysaccharides, it is of interest to determine the effect of PF on the capsular polysaccharide and extracellular polysaccharide of *S. suis*. The result shown in Figure 2f provided evidence that PF did not inhibit the production of CPS through LuxS/AI-2. In addition, as shown in Figure 2g, in the presence of PF, the production of EPS by *S. suis* was markedly reduced, even not significantly different from Δ*luxS*. Finally, according to previous results obtained by our research group, we selected *luxS* gene-regulated virulence genes to perform quantification by qPCR. The results showed that PF has a down-regulating effect on the transcription of these virulence genes, to reach levels comparable to those observed with Δ*luxS*. (Figure 2h). This finding provides direct evidence that the reduction in the production of EPS induced by the presence of PF is a key factor in the weakening of the biofilm matrix. In conclusion, these results strongly indicate that PF can affect the biofilm of *S. suis* through the LuxS/AI-2 system.

### Molecular interaction between PF and LuxS

**Figure 3.**
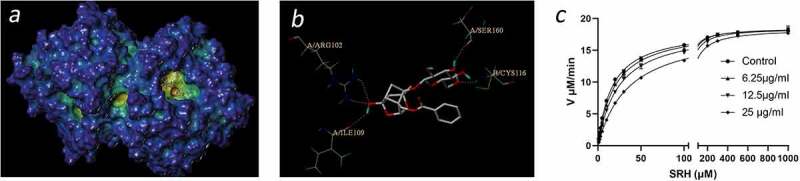
Interactions between PF on LuxS. (a) 3D structure of LuxS docked with PF. (b) Interactions between the binding site of PF and the amino acid residues of LuxS protein. (c) Inhibitory effect of PF on LuxS protein activity

The above results (Figure 2) suggest that PF can affect the LuxS/AI-2 system, but cannot inactivate the AI-2 signaling molecule. We thus analyzed the interaction between PF and LuxS using the Ellman method. As shown in Figure 3c, when the concentration of added PF increases, the Km value of LuxS gradually increases, although the maximum reaction rate does not change significantly, which indicates that PF is a competitive inhibitor of LuxS.

In order to further understand the interaction between PF and LuxS, we conducted a virtual docking experiment. As shown in (Figure 3a), the three-dimensional structure indicates that PF interacts with the LuxS active site and forms protein–ligand interactions with the key amino acid residues of LuxS. More specifically, the two-dimensional interaction map (Figure 3b) clearly shows that PF forms hydrogen bonds with ARG102, ILE109, SER160, and CYS116, respectively.

### Effect of PF in a mouse model of S. suis infection

**Figure 4.**
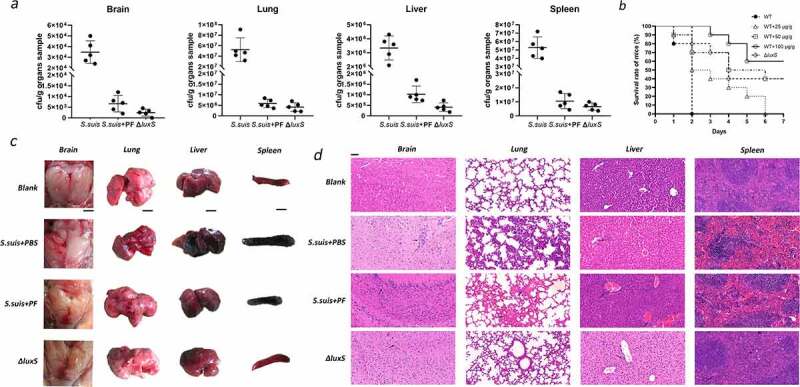
In vivo therapeutic effect of PF in a mouse model of *S. suis* infection. (a) Bacteria counts in brain, lung, liver, and spleen tissues. (b) Treatment effects of PF on *S. suis* HA9801 challenged mice. (c) The morphological changes of brain, lung, liver and spleen. Scale (black line): 1 cm. (d) Histopathology of *S. suis* infections caused. Arrows indicate histopathological changes in the HE staining of brain, lung, liver and spleen tissues. Magnification: 200 ×, Scale (black line): 50 μm. In Figures (a), (c) and (d), according to the body weight of the mice, the treatment group used 100 μg/g PF

In order to evaluate the potential therapeutic effect of PF as an inhibitor of the LuxS/AI-2 system, we used a mouse infection model. As shown in Figure 4b, PF showed a protective effect against *S. suis* HA9801 infection at doses of 25, 50, and 100 μg/g. Among them, PF at the doses of 50, 100 μg/g, the lethality of *S. suis* on mice was not significantly different from that of the Δ*luxS* group. From the dissection of the mouse organs (Figure 4c), it was found that treatment with PF resulted in almost no edema in the brain of the mice, while the lungs, liver, and spleen have milder lesions. The total bacterial count in brain, liver, spleen and lung (Figure 4a) of the PF-treated mouse group was significantly lower than that of control group, but there was no significant difference from the Δ*luxS* group. Histological analysis (Figure 4d) showed that there were no obvious brain lesions in the PF-treated group. Although the alveoli were slightly congested, there was inflammatory cell infiltration in the portal area of the liver, and moderate congestion in the spleen. However, compared with the untreated mouse group, the PF-treated group showed signs of remission. In addition, assessment of cell viability showed that PF (<100 μg/ml) was nontoxic (Figure S2) for human laryngeal epidermoid carcinoma cells (HEP-2). All the above clearly shows that PF may be an effective therapeutic agent to reduce *S. suis* infections by impairing the virulence of the bacterium.

## Discussion

*S. suis* type 2 is a highly pathogenic zoonotic pathogen [1] that causes serious economic losses in the pig industry, in addition to representing a serious threat to human life [3]. The strain HA9801 used in this study is a serotype 2 showing a typical high virulence and a biofilm-forming ability. Biofilms of *S. suis* bind to extracellular matrix proteins in both endothelial and epithelial cells and causes persistent infections. We previously identified nine unique proteins in the biofilm of *S. suis* through comparative proteomics analysis [34]. Further research found that the *pdh* significantly up-regulates the adhesion and invasion ability of *S. suis* [13], and the *otc* can improve the pathogenicity in the mouse abdominal infection model [15].

Our previous studies have shown that *S. suis* serotype 2 can regulate biofilm formation and virulence factor expression through the LuxS/AI-2 density sensing system, leading to a marked resistance to fluoroquinolones and tetracycline antibiotics [10,12,14,15,35]. Currently, there is little treatment option for infections caused by *S. suis* serotype 2, and consequently the search for antibiotic substitutes with the ability to inhibit bacteria in a biofilm state is an active field of research. Studies have found that sub-inhibitory concentrations of Syringopicroside [36] and Emodin [37] can effectively inhibit the formation of *S. suis* biofilm. In addition, the essential oils of cinnamon, thyme, and winter fragrant can also significantly inhibit the biofilm formation ability of *S. suis* [38]. In recent years, the therapeutic effects of various medicinal plants and natural plant compounds exhibiting anti-biofilm activities have attracted much attention [18]. In most studies, plant materials are used in the form of crude extracts, decorations or tinctures. Although these simple pharmaceutical preparations are often effective, their mechanisms of action are often not scientifically verified.

In this study, PF was found to significantly reduce the biofilm formation ability (Figure 1c) and virulence of *S. suis* at a concentration that does not affect the growth rate (Figure 1d). Notably, PF can reduce the production of AI-2 in *S. suis*, but it cannot inactivate AI-2 (Figure 2a). This shows that PF can directly or indirectly affect the biofilm formation ability and virulence of *S. suis* through the LuxS/AI-2 system. Moreover, the active site of PF is likely related to LuxS. In agreement with our observations, it was previously demonstrated that PF can affect *Candida albicans* (*C. albicans*) infection and inhibit the formation of carbapenemase-producing *Klebsiella pneumonia* (*K. pneumonia*) biofilm through QS system [24,39]. The three-dimensional structure of the biofilm of *S. suis* grown in the presence of sub-inhibitory concentrations of PF, as observed by scanning electron microscopy and laser confocal electron microscopy was found to be weakened. Biofilms are defined as aggregates of microorganisms embedded in polysaccharides secreted by them [40]. Therefore, we examined the effect of PF on the production of exopolysaccharides by *S. suis*. Previous reports have proved that bacterial polysaccharides are the matrix of bacterial biofilms [40]. The main components of the bacterial polysaccharides are divided into CPS and EPS. CPS are structural cell surface components of bacteria. Studies have shown that clinical pneumococcus encapsulated by CPS has impaired capacity to form biofilms [41]. The extracellular polysaccharide secreted in the mucilage can promote the adhesion between cells, thereby contributing to the formation of biofilm [42]. Further, we quantified the amounts of the extracellular polysaccharide produced by *S. suis*. The results showed that PF does not affect CPS content of cocci, but decrease the content of EPS. Obviously, the biofilm is probably affected by the weakening of EPS. This may be a mechanism by which PF can lead to the weakening of the *S. suis* biofilm. Our previous study showed that AI-2 overexpression [14] and deletion of *luxS* gene [10] can regulate some virulence factors of *S. suis*. In this study, PF can also regulate the transcription level of these virulence factors. Further studies are required to better determine how PF affects the biofilm and virulence of *S. suis*.

**Figure 5.**
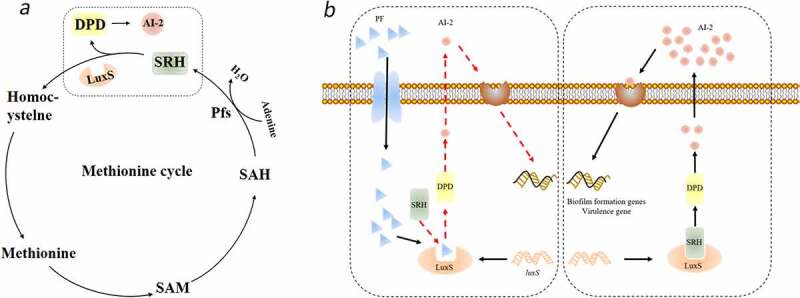
Proposed molecular mechanism underlying PF-induced attenuation of virulence and inhibition of biofilm formation in *S. suis*. (a) The circle is the methyl cycle pathway, the dashed frame is the pathway where the signal molecule AI-2 is generated. (b) The dashed box on the left is a diagram of the mechanism of PF affecting the formation of signaling molecule AI-2, and the dashed box on the right is a diagram of the mechanism of normal AI-2 signaling molecule formation

In this study, PF was found to affect the expression of AI-2 signaling molecules in *S. suis*. It is well known that the core of LuxS/AI-2 quorum sensing system is the AI-2 signal molecule synthetase LuxS. The enzyme is involved in the formation of HCY and DPD, and DPD forms AI-2 through self-cyclization, a furanone acyl boronic acid diester structure. AI-2 is actually a by-product of bacterial methyl metabolism (Figure 5a). Through the determination of the AI-2 concentration in *S. suis*, we found that PF does not affect the AI-2 signal molecules added to *S. suis*, but it can down-regulate the amount of AI-2 signal molecules secreted by *S. suis* itself. Therefore, the key enzyme involved in the regulation of the synthesis of AI-2 signal molecules-LuxS enzyme in *S. suis* is likely the target of PF (Figure 5b).

Our group previously purified the LuxS enzyme from *S. suis* type 2 and analyzed its structure in the early stage [25]. In this study, using a virtual molecular docking analysis, we demonstrated that PF may bind to LuxS enzyme, although further research is needed to further confirm these findings. We showed that PF can indeed inhibit LuxS enzyme activity, thus affecting the production of *S. suis* AI-2. Previous reports have proven that the quorum sensing system plays an important role in the formation of bacterial biofilms. Microorganisms use this information exchange, called Quorum Sensing (QS), to induce infectious diseases in eukaryotes, regulate their proliferation, and express their pathogenicity through QS, thereby evading the eukaryotic defense system. Further, the ability of PF to alleviate the symptoms of *S. suis* infections and to reduce colonization in vivo suggests broader applications aimed to prevent or treat *S. suis* associated infections (Figure 4).

In conclusion, our results indicate that PF interferes with the activity of the luxS enzyme in the LuxS/AI-2 quorum sensing system of *S. suis*, thereby reducing the secretion of EPS and attenuating the virulence, which ultimately leads to the decrease of pathogenicity. Therefore, PF may be used to guide the development of new anti-biofilm drugs to control *S. suis* infections.

## Supplementary Material

Supplemental Material

## Data Availability

The data used and/or analyzed during the current study are available from the corresponding author on reasonable request.
