# Supplementary material for: Paeoniflorin reduce luxS/AI-2 system-controlled biofilm formation and virulence in Streptococcus suis
Source: Virulence. 2021 Dec 18;12(1):3062–73. doi: 10.1080/21505594.2021.2010398 (PMC8923065; doi:10.1080/21505594.2021.2010398)
Supplement: Supplemental Material [file KVIR_A_2010398_SM4436.zip › supplementary/Supplementary materials 1.doc]

**Minimum Inhibitory Concentration (MIC) and Minimum Bactericidal Concentration (MBC)**

The broth dilution method was used to determine the minimum inhibitory concentration (MIC) and minimum bactericidal concentration (MBC) of the Paeoniflorin (PF). Briefly, PF were diluted to 6.4 mg/ml. Add 100 μl of PF in order that serial dilutions PF to 6.25, 12.5, 25, 50, 100, 200, 400, 800,1600, and 3200 μg/ml in 96-well plates containing 100 μl THB broth, in the final volume of 100 μl. Then, *S. suis* were added at 10^5^ CFU/ml to the 96 wells plate, in the final volume of 200 μl. Tests were performed in triplicates. The MIC was defined as the lowest extract concentration that showed no visible bacterial growth. 10 μl from each well showing no visible growth was cultured on THB agre. Resultant colonies were counted after an overnight incubation at 37 ℃. The minimal bactericidal concentration (MBC) was defined as the lowest concentration of antibiotic producing.

**Result**

The antibacterial activity of PF against *S. suis* was determined by determining the minimum inhibitory concentration (MIC) and the minimum bactericidal concentration (MBC), and the results are shown in Table S1. The MIC value and MBC value of PF were 400 μg/ml and ＞1600 μg/ml respectively.

**Table S1.** Minimal Inhibitory Concentration (MIC), Minimal Bactericidal Concentration (MBC), and the effect of PF against *S. suis* strains.

| Paeoniflorin | | |
| --- | --- | --- |
| Strain | MIC(μg/ml) | MBC(μg/ml) |
| HA9801 | 400 | ＞1600 |
