# Supplementary material for: Paeoniflorin reduce luxS/AI-2 system-controlled biofilm formation and virulence in Streptococcus suis
Source: Virulence. 2021 Dec 18;12(1):3062–73. doi: 10.1080/21505594.2021.2010398 (PMC8923065; doi:10.1080/21505594.2021.2010398)
Supplement: Supplemental Material [file KVIR_A_2010398_SM4436.zip › supplementary/Supplementary materials 2.doc]

**Table S1**. Primers for qPCR used in this study.

| Name | Sequence (5′-3′) | Target gene |
| --- | --- | --- |
| 16s RNA-S | GTTGCGAACGGGTGAGTAA | *16sRNA* |
| 16s RNA-A | TCTCAGGTCGGCTATGTATCG |  |
| Ccpa-S | CGGTGTCAGTGATATGGG | *ccpa* |
| Ccpa-A | GTCAGGTTTGGACGGGTA |  |
| Fbps-S | AACCATCTTGCCAGGCTCCAC | *fbps* |
| Fbps-A | CAGTTCAGAAGCCGTATCCCGAC |  |
| GapdH-S | CTTGGTAATCCCAGAATTGAACGG | *gapdH* |
| GapdH-A | TCATAGCAGCGTTTACTTCTTCAGC |  |
| Gdh-S | CACCTTTACCACCGCCGATTG | *gdh* |
| Gdh-A | GGAAATGTTCAAGTCAACCGTGG |  |
| Gor-S | GTTCACGCGCATCCTACG | *gor* |
| Gor-A | TACCAGGAATAGCAGGGAC |  |
| PerR-S | TTGAACACGTCATCCAACAT | *perR* |
| PerR-A | GTAGTTAGGTATTAGATCTTG |  |
