# Supplementary material for: Paeoniflorin reduce luxS/AI-2 system-controlled biofilm formation and virulence in Streptococcus suis
Source: Virulence. 2021 Dec 18;12(1):3062–73. doi: 10.1080/21505594.2021.2010398 (PMC8923065; doi:10.1080/21505594.2021.2010398)
Supplement: Supplemental Material [file KVIR_A_2010398_SM4436.zip › supplementary/Supplementary materials 3.doc]

**Determination of protein concentration by Bradford method**

Take 6 test tubes, number the test tubes according to the number designed in Table 1, and add the corresponding proportion of reagents to the corresponding numbered test tubes, and shake them evenly (Table S1). Add the prepared Coomassie Brilliant Blue Protein Detection Solution to each of the 6 test tubes. The added amount is 3.0 mL, shake evenly, and let stand for 3 minutes at room temperature. Use a visible light spectrophotometer to determine A595. As shown in Table 1, tube 1 is Blank control. The ordinate is A595, the abscissa is the standard protein content, and the standard protein concentration curve is made (Fig. S1).

Then add 80 µL of double-distilled water to 20 µL LuxS protein concentrate, shake and mix well. Then add 3.0 mL of Coomassie Brilliant Blue Protein Detection Solution, shake and mix, let stand at room temperature for 3 minutes, and measure the absorbance at A595. Repeat the experiment three times, take the average value, and calculate the LuxS protein concentration by referring to the above standard curve.

**Table S1** Standard protein solution

| Test tube number | | 1 | 2 | 3 | 4 | 5 | 6 |
| --- | --- | --- | --- | --- | --- | --- | --- |
| V (Standard protein) | | 0μL | 20μL | 40μL | 60μL | 80μL | 100μL |
| V(Double distilled water) | | 100μL | 80μL | 60μL | 40μL | 20μL | 0μL |
| Protein concentration | 0mg/mL | | 0.2mg/mL | 0.4mg/mL | 0.6mg/mL | 0.8mg/mL | .1mg/mL |


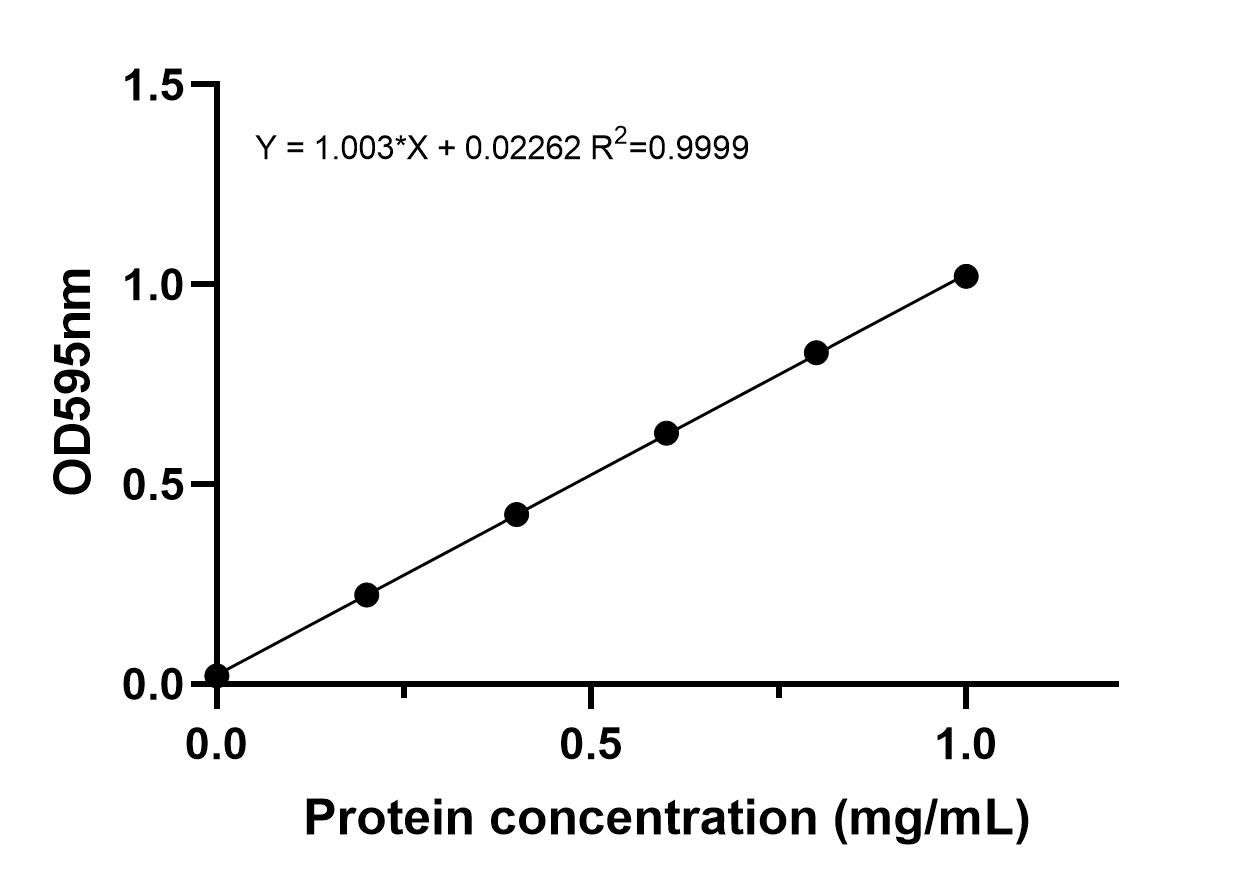


**Fig. S1 Standard protein curve.**
