# Supplementary figures and images for: Paeoniflorin reduce luxS/AI-2 system-controlled biofilm formation and virulence in Streptococcus suis
Source: Virulence. 2021 Dec 18;12(1):3062–73. doi: 10.1080/21505594.2021.2010398 (PMC8923065; doi:10.1080/21505594.2021.2010398)

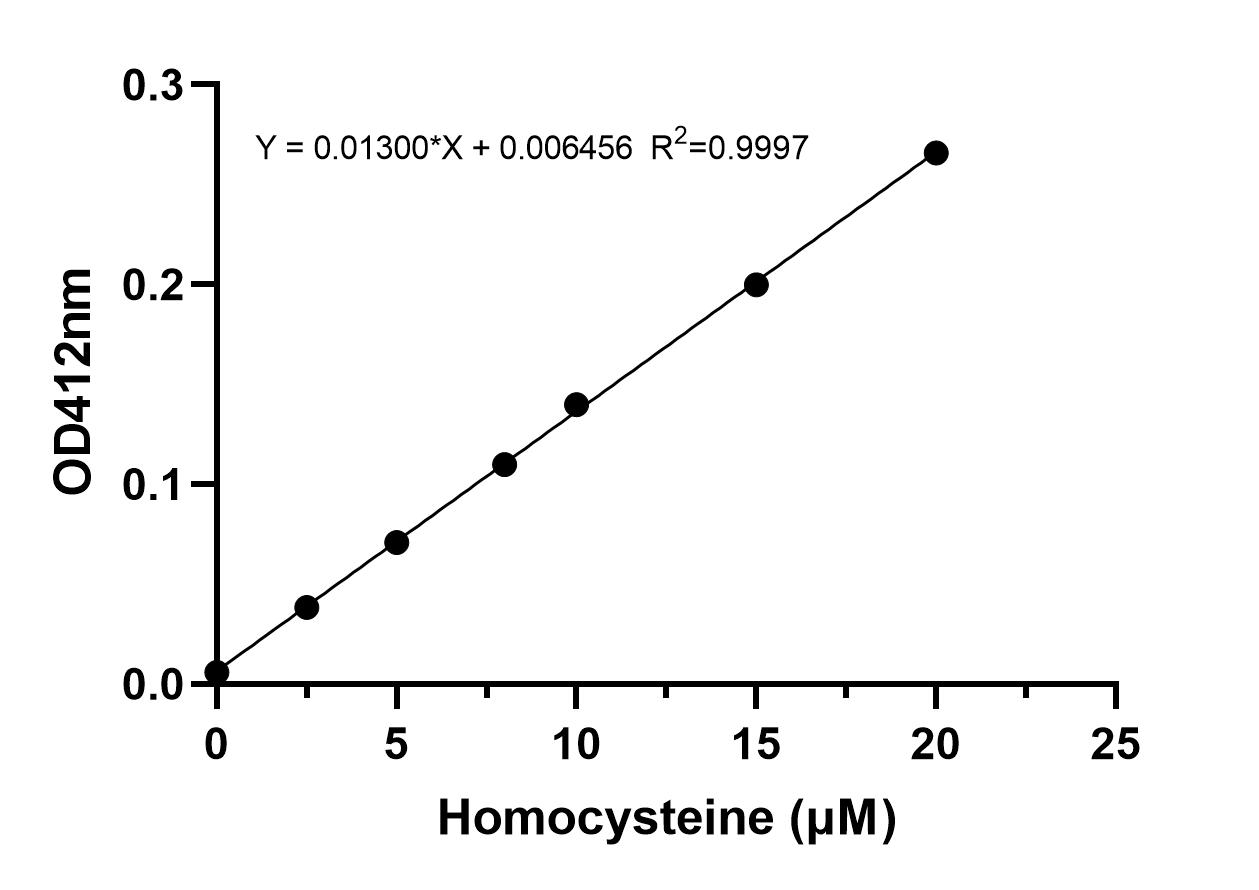

Supplement: Supplemental Material [file KVIR_A_2010398_SM4436.zip › supplementary/Figure S1.jpg]

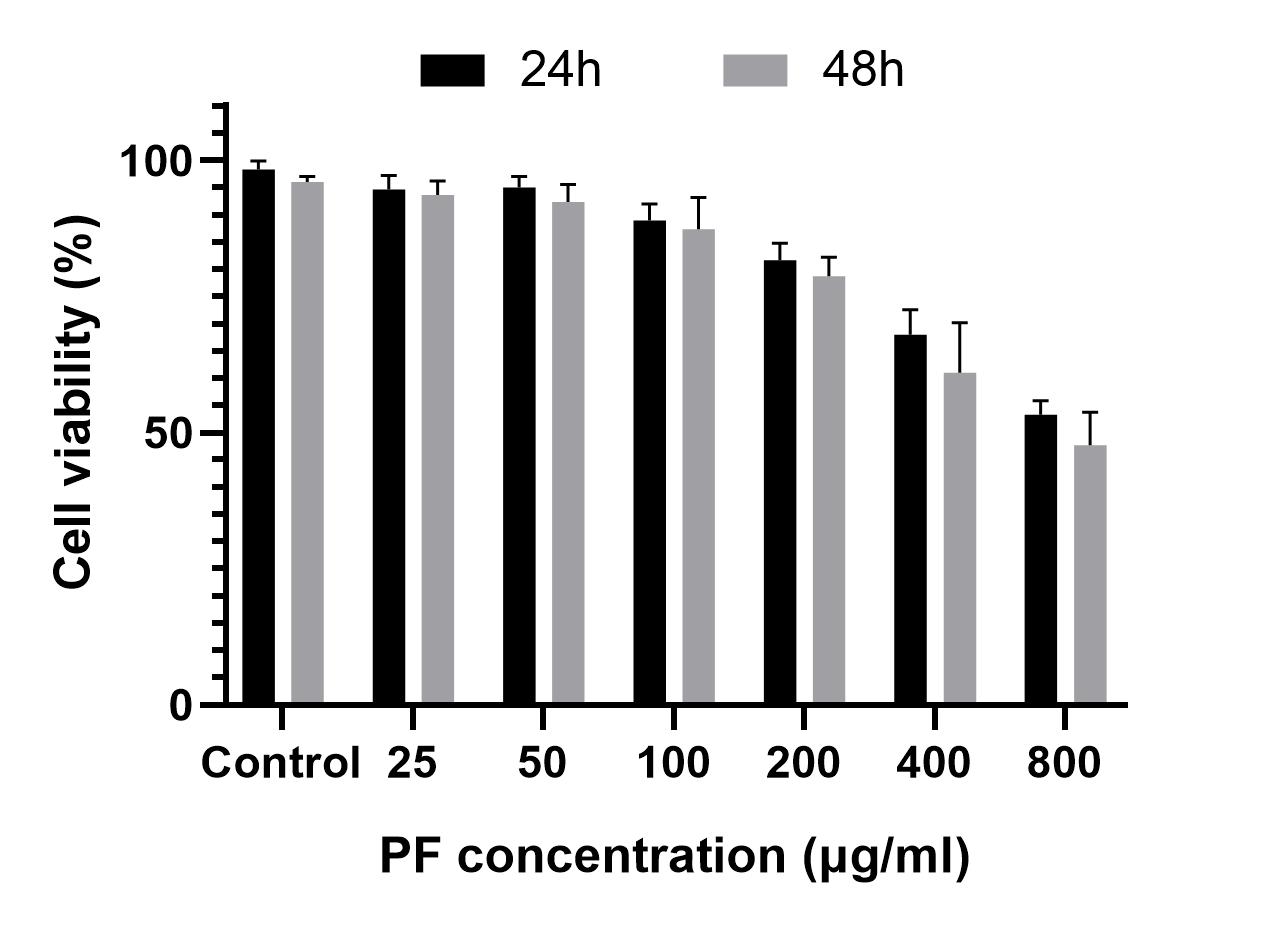

Supplement: Supplemental Material [file KVIR_A_2010398_SM4436.zip › supplementary/Supplementary materials 5, Fig. S2.jpg]
